# Evaluation of relationship between endoscopic activity index and inflammatory indicators such as fecal calprotectin, C-reactive protein to albumin ratio, neutrophil to lymphocyte ratio, platelet to lymphocyte ratio in ulcerative colitis patients

**DOI:** 10.5937/jomb0-58955

**Published:** 2026-01-28

**Authors:** Tarang Taghvaei, Hashemi Seyyed Abbas, Iradj Maleki, Mahboobe Ebrahimi, Arash Kazemi, Shahabi Reyhane Ebrahimi, Charati Jamshid Yazdani

**Affiliations:** 1 Mazandaran University of Medical Sciences, Gut and Liver Research Centre, Sari, Iran; 2 Mazandaran University of Medical Sciences, Gut and Liver Research Centre, Non-Communicable Diseases Institute, Sari, Iran, Department of Microbiology, Ayatollah Amoli Branch, Islamic Azad University, Amol, Iran; 3 Mazandaran University of Medical Sciences, Department of Biostatistics, Health Sciences Research Centre, Addiction Institute, Sari, Iran

**Keywords:** ulcerative colitis, biomarkers, platelet-to-lymphocyte ratio, neutrophil-to-lymphocyte ratio, faecal calprotectin, c-reactive protein, disease severity, ulcerozni kolitis, biomarkeri, odnos trombocita i limfocita, odnos neutrofila i limfocita, fekalni kalprotektin, C-reaktivni protein, težina bolesti

## Abstract

**Background:**

This study aimed to evaluate the association between low-cost inflammatory biomarkers and disease severity in ulcerative colitis (UC), with a focus on predicting acute severe ulcerative colitis (ASUC) and active disease (Mayo score &gt;1).

**Methods:**

An analytical cross-sectional study was conducted on 131 patients diagnosed with UC at Imam Khomeini Hospital, Iran, between 2022 and 2024. Demographic, clinical, and laboratory data - including neutrophil-to-lymphocyte ratio (NLR), platelet-to-lymphocyte ratio (PLR), CRP-to-albumin ratio (CAR), and faecal calprotectin - were collected. Disease activity was evaluated using the Mayo Endoscopic Score (MES). Statistical analyses included chi-square tests, ANOVA, and multivariable logistic regression adjusted for age, sex, and body mass index (BMI).

**Results:**

The mean age of participants was 45.8± 15.9 years. Elevated inflammatory markers were observed (CRP: 39.1 ± 32.6 mg/L; ESR: 44 .0± 21.5 mm/hr). Defecation frequency S6/day was significantly associated with ASUC (c2(2) = 101.10, p&lt; 0.001). After adjustment, PLR in the third quartile (14-20) was independently associated with ASUC (O R = 2.57, p= 0.034). The percentage of monocytes was significantly higher in ASUC patients (F(1, 30) = 6.52, p= 0.016). No significant associations were found for NLR or CAR. PLR also differed significantly between active and inactive UC groups (median [ IQR] : 133.87 [96.15-190.63] vs. 129.61 [102.15-209.98], p= 0.029), although its discriminatory power was limited.

**Conclusions:**

PLR and monocyte percentage may serve as accessible indicators for assessing UC severity and identifying patients at risk for ASUC. These findings support the supplementary use of routine inflammatory blood indices in the management of UC, especially in resource-limited settings.

## Abstract (sr)

**Uvod:**

Ova studija je imala za cilj da proceni povezanost niskobudžetnih inflamatornih biomarkera sa težinom bolesti kod pacijenata sa ulceroznim kolitisom (UK), sa posebnim fokusom na predviđanje akutnog teškog ulceroznog kolitisa (ATUK) i aktivne bolesti (Mayo skor &gt;1).

**Metode:**

Analitička studija preseka je sprovedena na 131 pacijentu sa dijagnozom UK u bolnici Imam Homeini, Iran, u periodu od 2022. do 2024. godine. Prikupljeni su demografski, klinički i laboratorijski podaci, uključujući odnos neutrofila i limfocita (NLR), odnos trombocita i limfocita (PLR), odnos CRP-a i albumina (CAR) i fekalni kalprotektin. Aktivnost bolesti je procenjivana pomoću Mayo endoskopskog skora (MES). Statističke analize uključivale su hi-kvadrat test, ANOVA i multivarijantnu logističku regresiju prilagođenu za uzrast, pol i indeks telesne mase (BMI).

**Rezultati:**

Prosečna starost učesnika je bila 45,8± 15,9 godina. Zabeleženi su povišeni inflamatorni markeri (CRP: 39,1 ± 32,6 mg/L; SE: 44,0± 21,5 mm/h). Učestalost defekacije S6 puta dnevno bila je značajno povezana sa ATUK (c2(2) = 101,10, p &lt; 0,001). Nakon prilagođavanja, PLR u trećem kvartilu (14-20) bio je nezavisno povezan sa ATUK (O R = 2,57, p= 0,034). Procenat monocita bio je značajno viši kod pacijenata sa ATUK (F(1,30) = 6,52, p = 0,016). Nisu pronađene značajne povezanosti za NLR ili CAR. PLR se takođe značajno razlikovao između aktivne i neaktivne UK grupe (medijana [IQR]: 133,87 [96,15-190,63] vs. 129,61 [102,15-209,98], p = 0,029), iako je njegova diskriminatorna moć bila ograničena.

**Zaključak:**

PLR i procenat monocita mogu predstavljati dostupne pokazatelje za procenu težine UK i identifikaciju pacijenata sa rizikom za ATUK. Ovi nalazi govore u prilog upotrebi rutinskih inflamatornih krvnih indeksa u upravljanju UK, posebno u uslovima sa ograničenim resursima.

## Introduction

Inflammatory bowel diseases (IBD), including ulcerative colitis (UC) and Crohn's disease (CD) are chronic, relapsing inflammatory conditions of the gastrointestinal tract. These disorders share overlapping symptoms such as diarrhoea, abdominal pain, weight loss, and rectal bleeding [1] [2]. Among them, UC is more common and primarily affects the colon and rectum, with patients typically presenting with increased stool frequency, faecal urgency, rectal bleeding, and abdominal discomfort [3].

UC is the most prevalent form of IBD worldwide, with an estimated global burden of approximately 5 million cases in 2023 [4]. The disease begins in the rectum and may extend proximally throughout the colon. Endoscopic evaluation remains a key tool for diagnosis and monitoring. Typical endoscopic findings in UC include mucosal erythema, oedema, friability, and loss of vascular pattern. However, visual interpretation is subjective and requires standardisation [5] [6] [7]. The Mayo Endoscopic Score (MES) is the most commonly used system to assess UC severity due to its simplicity and reproducibility [8].

In clinical practice, various laboratory markers such as C-reactive protein (CRP), erythrocyte sedimentation rate (ESR), white blood cell count (WBC), haemoglobin (Hb), and albumin (ALB) are used to estimate disease activity [9]. Faecal calprotectin is a non-invasive marker that reliably reflects intestinal inflammation and correlates well with endoscopic findings [10] [11]. CRP, though nonspecific, is also widely used and correlates with moderate to severe disease [12]. Faecal calprotectin is especially valuable due to its sensitivity, low cost, and convenience in monitoring disease activity [13] [14] [15].

Recently, inflammatory ratios derived from complete blood count (CBC) tests, such as the neutrophil-to-lymphocyte ratio (NLR), platelet-to-lymphocyte ratio (PLR), and CRP-to-albumin ratio (CAR), have gained attention as accessible and cost-effective biomarkers. NLR has been found to reflect systemic inflammation and UC activity [16]. At the same time, PLR is increasingly studied for its role in disease assessment, given the involvement of both platelets and lymphocytes in the pathogenesis of UC [17] [18]. Similarly, CAR combines the inflammatory response and nutritional status and may outperform CRP alone in predicting severe UC [19].

Despite encouraging preliminary findings, the reliability and consistency of these markers in predicting endoscopic severity and acute severe UC (ASUC) remain unclear. Small sample sizes and inconsistent methodologies limit the majority of existing studies.

Therefore, the present study aims to evaluate the association between NLR, PLR, CAR, and faecal calprotectin with endoscopic disease severity, as determined by the Mayo scoring system, and the prediction of ASUC. Given the low cost and minimal invasiveness of these markers, they could serve as valuable tools in resource-limited settings for assessing disease activity and guiding treatment decisions.

## Materials and methods

### Study design and setting

This analytical cross-sectional study was conducted between 2022 and 2023 at Imam Khomeini Hospital, affiliated with Mazandaran and Shahid Beheshti Universities of Medical Sciences, Iran. The study targeted patients diagnosed with ulcerative colitis (UC) who were referred to liver and gastrointestinal clinics during this period.

### Participants

A total of 131 patients with confirmed UC were included through convenience sampling. The inclusion criteria comprised adult patients (≥18 years) with a confirmed diagnosis of UC, based on clinical, endoscopic, and histological findings. Patients were excluded if they had incomplete medical records, declined participation, or had any of the following conditions: prior gastrointestinal surgery, other autoimmune or hematological diseases, malignancies, significant comorbidities (e.g., cardiovascular or chronic kidney disease), systemic infections, or recent/current use of medications such as hormonal contraceptives, anticoagulants, or antiplatelet agents (e.g., estrogen, progesterone, heparin).

### Ethical considerations

Ethical approval was obtained from the Ethics Committee of Mazandaran University of Medical Sciences (Code: I R.MAZUMS. IMAM HOSPITAL.REC.1403.025). Written informed consent was obtained from all participants prior to enrollment.

### Data collection and variables

Demographic data (age, sex, body mass index), clinical characteristics, and laboratory parameters were collected. Inflammatory biomarkers assessed included:

- Neutrophil-to-lymphocyte ratio (NLR)
- Platelet-to-lymphocyte ratio (PLR)
- CRP-to-albumin ratio (CAR)
- C-reactive protein (CRP)
- Erythrocyte sedimentation rate (ESR)
- Faecal calprotectin

All biomarkers were derived from complete blood counts, standard blood tests, and stool analysis. Endoscopic evaluation was performed by two experienced gastroenterologists using the Mayo Endoscopic Score (MES) (range: 0-3). Disease extent was classified according to the Montreal classification:

- E1: Ulcerative proctitis
- E2: Left-sided colitis (up to the splenic flexure)
- E3: Extensive colitis (beyond the splenic flexure)
- Symptom severity was graded as:
- S0: Asymptomatic<br>S1: Mild<br>S2: Moderate
- S3: Severe (≥6 bloody stools/day, fever, tachycardia, anaemia, ESR >30 mm/hr)

### Definition of outcomes

#### Active disease: Defined as MES ≥2

Acute Severe Ulcerative Colitis (ASUC): Defined as ≥6 bloody stools/day plus at least one of the following: fever (>37.8°C), tachycardia (>90 bpm), haemoglobin <10.5 g/dL, or ESR >30 mm/hr.

### Statistical analysis

Data were analysed using Stata version 17. Normality of continuous variables was assessed with the Kolmogorov-Smirnov test. Descriptive statistics included means ± standard deviation (SD) for continuous variables and frequencies (%) for categorical variables.

Group comparisons were conducted using the following methods: a chi-square test for categorical variables and one-way analysis of variance (ANOVA) for continuous variables, with post-hoc Tukey tests for multiple comparisons.

Logistic regression analysis was used to examine the association between inflammatory markers and ASUC, adjusting for age, sex, and BMI. The diagnostic performance of PLR, NLR, CAR, and CRP for ASUC was evaluated using receiver operating characteristic (ROC) curves, with area under the curve (AUC) and 95% confidence intervals reported. A *p*-value <0.05 was considered statistically significant.

## Results

### Patient characteristics

The study enrolled 131 patients with ulcerative colitis (UC), consisting of 57 males (43.5%) and 74 females (56.5%), with a mean age of 45.8±15.9 years. The average disease duration was 6.2±5.6 years. Laboratory tests showed a mean haemoglobin level of 11.3±2.1 g/dL, elevated C-reactive protein (CRP) at 32.6±39.1 mg/L, and high faecal calprotectin averaging 564.4±374.1 μg/g. Most patients exhibited moderate to severe endoscopic activity (72.1% Mayo II-III), with left-sided colitis (E2) being the most common disease extent (51.9%). Stool frequency was predominantly 4-6 times per day (46.8%). Common comorbidities were diabetes (19.8%) and hypertension (12.2%). The most commonly prescribed medications were mesalazine (59.5%) and azathioprine (38.9%).

Colonoscopic findings revealed widespread mucosal inflammation, characterised by erythema (53.4%), ulcers (43.5%), erosions (42.7%), spontaneous bleeding (14.5%), and loss of vascular pattern (12.2%). Only 3.1% had a normal colonoscopy.

### ASUC associations

Among the 131 patients, 15 had acute severe ulcerative colitis (ASUC) and 116 did not. Defecation frequency was strongly associated with ASUC status (χ^2^(2) = 101.10, *p*< .001), with all ASUC cases reporting stool frequency ≥6 times/day. No significant associations were found between ASUC and gender, diabetes, or hypertension. Table 1

**Table 1:** Baseline characteristics of study subjects.

Variable	Mean±SD/<br>n (%)	Variable	Mean±SD/<br>n (%)	Variable	Mean±SD/<br>n (%)
**Gender**		**ESR (mm/hr)**	44.04±21.47	**Medications**	
**- Male**	57 (43.5%)	**Faecal Calprotectin**<br>**(mg/g)**	564.35±374.06	- Mesalazine	78 (59.5%)
**- Female**	74 (56.5%)	PLR	254.42±574.6	- Azathioprine	51 (38.9%)
**Age (years)**	45.82±15.93<br>(15-87)	**Mayo Endoscopic**<br>**Score**		- Infliximab	15 (11.5%)
**Height (cm)**	167.52±9.12	- Mild (Mayo I)	27.9%	- Adalimumab	12 (9.2%)
**Weight (kg)**	72.05±12.62	- Moderate (Mayo II)	25.4%	- Steroids	10 (7.6%)
**BMI**	25.59±3.93	- Severe (Mayo III)	46.7%	- Tofacitinib	1 (0.8%)
**Disease Duration**<br>**(years)**	6.17±5.56	**Daily Stool Frequency**		**ColonoscopicFindings**	
**Haemoglobin (g/dL)**	11.33±2.14	- ≤4 times/day	42 (38.5%)	- Mucosal erythema	70 (53.4%)
**WBC (×10^3^/mL)**	11.90±10.29	- 4-6 times/day	51 (46.8%)	- Ulcers	57 (43.5%)
**Platelets (×10^3^/mL)**	336.50±126.68	- ≥6 times/day	16 (14.7%)	- Erosions	56 (42.7%)
**Neutrophils (%)**	64.66±15.36	**Disease Extent (Colon)**		- Fragility	23 (17.6%)
**Lymphocytes (%)**	24.21±10.16	- E1 (Rectum only)	43 (33.3%)	- Spontaneous<br>bleeding	19 (14.5%)
**Monocytes (%)**	6.90±4.26	- E2 (Left colon)	67 (51.9%)	- Loss of vascular<br>pattern	16 (12.2%)
**CRP (mg/L)**	32.58±39.14	- E3 (Extensive)	19 (14.7%)	- Normal<br>colonoscopy	4 (3.1%)

**Figure 1:**
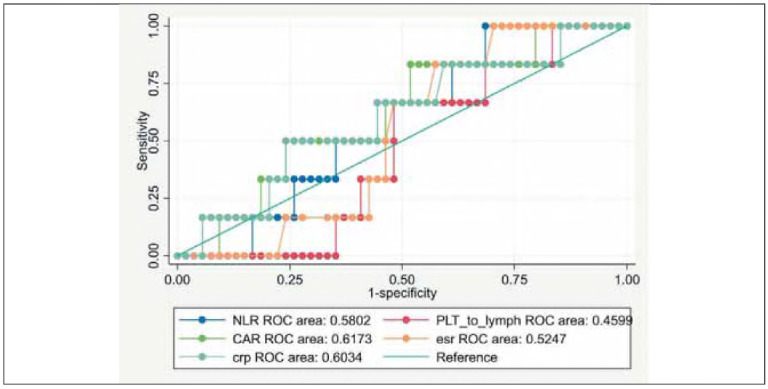
Sensitivity and specificity of markers.

ANOVA revealed no significant differences in age, BMI, or most inflammatory markers, except for a significantly lower monocyte percentage in ASUC patients (*p* = 0.016). The platelet-to-lymphocyte ratio (PLR) did not differ significantly between groups overall; however, quartile analysis revealed that ASUC cases were overrepresented considerably in the third PLR quartile (*p*= .028) (Figure 1).

### Logistic regression of PLR quartiles on ASUC risk

After adjusting for age, sex, and BMI, patients in the third PLR quartile had significantly higher odds of ASUC (OR=2.57, *p*= .034) compared to those in the first quartile (reference group). The second quartile was not significantly associated with ASUC risk. Table 2 Table 3

**Table 2:** Comparison between ASUC and non-ASUC patients.

Variable	Category	No ASUC (n = 116)	ASUC (n = 15)	Statistic	p-value
Gender	Male	53 (45.7%)	4 (26.7%)	χ^2^(1) = 1.96	0.162
	Female	63 (54.3%)	11 (73.3%)		
Age (years)	Mean (SD)	45.77 (16.16)	46.20 (14.63)	F(1,127)=0.01	0.922
BMI	Mean (SD)	25.74 (4.08)	24.39 (2.33)	F(1,82)=0.95	0.333
Diabetes (DM)	Yes	23 (19.8%)	3 (20.0%)	χ^2^(1)<0.01	0.987
	No	93 (80.2%)	12 (80.0%)		
Hypertension (HTN)	Yes	13 (11.2%)	3 (20.0%)	χ^2^(1)=0.96	0.328
	No	103 (88.8%)	12 (80.0%)		
Defecation<br>Frequency	≤4 times/day	42 (44.7%)	0 (0.0%)	χ^2^(2)=101.10	<.001
	4-6 times/day	51 (54.3%)	0 (0.0%)		
	≥6 times/day	1 (1.1%)	15 (100.0%)		
Extent (E1/E2/E3)	E1	39 (34.2%)	4 (26.7%)	χ^2^(2)=0.46	0.794
	E2	58 (50.9%)	9 (60.0%)		
	E3	17 (14.9%)	2 (13.3%)		
Monocytes (%)	Mean (SD)	7.48 (4.06)	1.39 (1.05)	F(1,30)=6.52	0.016
PLR	Median (IQR)	140.46 (97.8-210.1)	113.13 (95.67-164.59)	U = 643, z=1.20	0.233
PLR Quartiles	Q1	25 (26.0%)	3 (21.4%)	χ^2^(3)=9.10	0.028
	Q2	25 (26.0%)	2 (14.3%)		
	Q3	20 (20.8%)	8 (57.1%)		
	Q4	26 (27.1%)	1 (7.1%)		

**Table 3:** Logistic regression analysis of PLR quartiles and ASUC risk. * Statistically significant

PLR Quartile	Coefficient (β)	Std. Error	z-value	p-value	95% CI Lower	95% CI Upper
1 (Reference)	-	-	-	-	-	-
2	1.173	1.322	0.89	0.375	-1.419	3.765
3	2.575*	1.215	2.12	0.034	0.194	4.956

### Comparison between active and non-active UC patients

Patients with active UC (n = 82) exhibited a marginally higher erythrocyte sedimentation rate (ESR) than those with non-active disease (n = 25) (46.39± 19.84 mm/hr vs. 37.00±24.88 mm/hr; t(98) = -1.92, *p*= .057). The platelet-to-lymphocyte ratio (PLR) was significantly different between active and non-active groups (*p*< .05), though it had limited diagnostic accuracy (AUC=0.42). No significant differences were found for neutrophil-to-lymphocyte ratio (NLR), CRP-to-albumin ratio (CAR), or CRP (Table 4).

**Table 4:** Comparison of inflammatory markers by disease activity.

Marker	Active UC (n=82)	Non-Active UC	t-value	p-value	AUC (95% CI)
ESR (mm/hr)	46.39±19.84	37.00±24.88	-1.92	0.057	-
PLR	133.87 (median)	129.61 (median)	-	<0.05	0.42 (0.30-0.54)
NLR	-	-	-	>0.05	0.51 (0.39-0.63)
CAR	-	-	-	>0.05	0.52 (0.41-0.63)
CRP (mg/L)	-	-	-	>0.05	0.58 (0.46-0.69)

## Discussion

This study identified CRP as a significant biomarker associated with UC severity, particularly in distinguishing between severe and mild-to-moderate cases of UC. This reinforces the clinical utility of CRP as a widely available, cost-effective marker for inflammation in UC, making it useful for both initial assessment and monitoring. However, other inflammatory indices, such as NLR, PLR, CAR, and faecal calprotectin, did not demonstrate consistent correlations with disease severity or ASUC in our cohort. These findings underscore the complexity of UC pathophysiology and the limitations of single biomarkers in capturing the multifaceted nature of the inflammatory processes involved.

Several prior studies have reported more promising roles for NLR and PLR in assessing UC activity. For instance, NLR has been suggested as a reliable, inexpensive marker reflecting systemic inflammation and disease activity [20]. Similarly, PLR has been linked to disease severity and used in conjunction with faecal calprotectin to enhance the detection of endoscopic remission [21]. In addition, CAR has been proposed as a highly sensitive marker, sometimes outperforming CRP in predicting severe UC [22] [23]. The discrepancies with our findings may be attributed to the limited sample size, potential selection bias, or differences in disease characteristics and treatment regimens in our population.

Faecal calprotectin is widely accepted as a sensitive and non-invasive biomarker of intestinal inflammation and mucosal healing, with numerous studies validating its utility in the management of UC [24] [25]. Its lack of significant association with disease severity in our study may be due to the small subset of patients with available calprotectin data and variations in disease phase or treatment at the time of sampling. Previous longitudinal studies have demonstrated that faecal calprotectin levels correlate with endoscopic disease activity and can predict relapse and response to therapy [26] [27]. Furthermore, faecal calprotectin has been shown to outperform CRP in specific settings, underscoring the importance of combining systemic and faecal markers for a comprehensive assessment [28].

The role of systemic inflammatory markers such as CRP remains complex. While elevated CRP levels generally correlate with active inflammation, some studies suggest that CRP levels may not always accurately reflect mucosal healing or clinical remission, particularly in patients with limited colonic involvement. This may relate to the heterogeneity of UC in terms of disease extent and individual immune response profiles. Additionally, emerging evidence suggests that CRP may function more as an acute-phase reactant reflecting systemic inflammation rather than a specific mediator of colonic inflammation [29].

Our findings are in line with reports that found no significant correlation between NLR and disease extent or severity [28]. Similarly, animal model studies demonstrate that CRP does not directly influence disease phenotype or biochemical markers, suggesting a secondary role in inflammation [28]. Other haematological parameters, such as red cell distribution width (RDW) and platelet counts, have also yielded inconsistent results in predicting disease activity [30] [31] [32].

Interestingly, the observation that extremely high PLR values did not correspond to an increased risk of ASUC suggests a complex immunological interplay. Platelets are increasingly recognised as active participants in both pro-inflammatory processes and tissue repair mechanisms [33] [34]. Lymphocytes similarly play a dual role, balancing immune activation and regulation. This duality may explain the non-linear relationship observed, suggesting that PLR alone may not be sufficient to capture the disease's dynamics. Further mechanistic studies on platelet-lymphocyte interactions in intestinal inflammation could open avenues for novel biomarker development and targeted therapies.

Moreover, other biomarkers have gained attention in recent UC research. For example, serum cytokines such as IL-6, TNF-alpha, and calprotectin-derived proteins have shown promise in reflecting disease activity and predicting outcomes [35]. Emerging multi-omics approaches, which combine genetic, proteomic, and metabolomic data, aim to create comprehensive biomarker panels that may overcome the limitations of single markers [36]. Our study's findings emphasise the need for integrated biomarker models that incorporate clinical, laboratory, and endoscopic parameters to improve diagnostic and prognostic accuracy.

This study has several limitations that should be considered when interpreting the findings. First, the cross-sectional design limits the ability to establish causal relationships or evaluate changes in biomarker levels over time. Second, the relatively small sample size, particularly for faecal calprotectin measurements, reduces statistical power and may contribute to Type II errors, thereby limiting the detection of subtle but clinically relevant associations. Third, incomplete availability of biomarker data across the patient cohort introduces potential selection bias and restricts comprehensive analysis. Fourth, variability in patient treatment regimens and disease duration was not fully controlled, which may have influenced biomarker levels and disease severity assessments. Lastly, the single-centre nature of the study may limit the generalizability of the results to broader, more diverse populations with ulcerative colitis.

Future studies should focus on larger, longitudinal cohorts to validate the performance of biomarkers and clarify their roles in predicting disease course and complications. Developing combined biomarker panels using machine learning and artificial intelligence may enhance risk stratification and individualised patient management. Additionally, investigating the biological roles of platelets, lymphocytes, and other immune cells in mucosal inflammation and repair is crucial for identifying novel therapeutic targets.

## Conclusion

Overall, this study suggests that although some inflammatory markers, such as CRP and PLR, are somewhat associated with UC severity, none alone possesses sufficient sensitivity and specificity to accurately predict acute severe UC. The unexpected PLR findings underscore the complexity of UC pathophysiology and the need for combined predictive algorithms that incorporate inflammatory, clinical, and endoscopic markers. This research opens up avenues to explore the roles of platelets and lymphocytes in intestinal inflammation and repair, paving the way for future therapeutic strategies. Larger, longitudinal studies are needed to define optimal biomarker combinations to predict severe UC complications more reliably.

## Dodatak

### Acknowledgments

We would like to thank all the patients who participated in this study sincerely. We also appreciate the valuable contributions of our clinical and laboratory staff who assisted with data collection and analysis.

### Conflict of interest statement

All the authors declare that they have no conflict of interest in this work.
